# The Dairy Matrix: Its Importance, Definition, and Current Application in the Context of Nutrition and Health

**DOI:** 10.3390/nu16172908

**Published:** 2024-08-31

**Authors:** Ana-Isabel Mulet-Cabero, Moises Torres-Gonzalez, Jan Geurts, Ashley Rosales, Bita Farhang, Corinne Marmonier, Ellen Kathrine Ulleberg, Erica Hocking, Isabelle Neiderer, Ivana Gandolfi, Laura Anderson, Lea Brader, Maretha Vermaak, Melissa Cameron, Merete Myrup Christensen, Rivkeh Haryono, Stephan Peters

**Affiliations:** 1 International Dairy Federation, 1030 Brussels, Belgium; 2 National Dairy Council, Rosemont, IL 60018-5616, USA; moises.torres-gonzalez@dairy.org; 3 FrieslandCampina, 3818 LE Amersfoort, The Netherlands; jan.geurts@frieslandcampina.com; 4 Dairy Council of California, Sacramento, CA 95834, USA; arosales@dairycouncilofca.org; 5 Dairy Farmers of Ontario, Mississauga, ON L5N 2L8, Canada; 6 Centre National Interprofessionnel de l’Economie Laitière C.N.I.E.L., 75009 Paris, France; 7 Norwegian Dairy Council, 0650 Oslo, Norway; ellen@melk.no; 8 Dairy UK, London WC1V 7EP, UK; ehocking@dairyuk.org; 9 Dairy Farmers of Canada, Montreal, QC H3B 2N2, Canada; isabelle.neiderer@dfc-plc.ca; 10 Parmalat, 43044 Parma, Italy; 11 Fonterra Co-Operative Group Ltd., Auckland 1010, New Zealand; laura.anderson@fonterra.com; 12 Arla Foods, 8200 Aarhus, Denmark; lebrd@arlafoods.com; 13 Milk SA, Pretoria 0081, South Africa; maretha@dairycep.co.za; 14 Dairy Australia, Melbourne, VIC 3006, Australiarivkeh.haryono@dairyaustralia.com.au (R.H.); 15 Danish Agriculture and Food Council, 1609 København V, Denmark; 16 Nederlandse Zuivel Organisatie, 2596 BC The Hague, The Netherlands; peters@nzo.nl

**Keywords:** dairy matrix, food matrix, dairy foods, food structure, foods, dairy matrix’s health effects, nutrition, human health

## Abstract

Nutrition research has shifted from single nutrients to examining the association of foods and dietary patterns with health. This includes recognizing that food is more than the sum of the individual nutrients and relates to the concept of the food matrix. Like other foods, dairy foods are characterized by their unique matrices and associated health effects. Although the concepts of the food matrix and/or dairy matrix are receiving increasing attention in the nutrition and health literature, there are different terms and definitions that refer to it. This article aims to provide insights into the application of the concepts of the food matrix and dairy matrix and to provide a current overview of the definitions and terminology surrounding the food matrix and dairy matrix. By analysing these aspects, we aim to illustrate the practical implications of the food matrix and dairy matrix on nutrition and health outcomes and evaluate their roles in shaping evidence-based policies for the benefit of public health. There is a need for harmonized definitions within the literature. Therefore, the International Dairy Federation put forward harmonized terms to be internationally applicable: the “dairy matrix” describes the unique structure of a dairy food, its components (e.g., nutrients and non-nutrients), and how they interact; “dairy matrix health effects” refers to the impact of a dairy food on health that extend beyond its individual components.

## 1. From Nutrients to Foods: The Emergence of the Food Matrix

Nutrition research has traditionally focused on identifying the specific association through which individual nutrients impact health outcomes, for example, vitamin C for the prevention of scurvy, calcium and its importance for bone health, and the association of saturated fats with a risk of cardiovascular diseases (CVDs). This approach, which focuses on the health effects of individual nutrients, has been described as a reductionist and narrowing perspective, as people do not consume nutrients in isolation but as part of a food [1].

In the last half-century, as remarked by the Food and Agriculture Organization of the United Nations and the World Health Organization [2], the focus of nutrition research has evolved and shifted to examining the association of foods and dietary patterns with health [3], which is mainly achieved through epidemiological studies. Epidemiological food research examines the relationship between the intake of foods and health outcomes, such as non-communicable diseases (NCDs) and health risk factors. The shift from nutrient-focused to food-focused research acknowledges that the health effects of foods are not solely determined by individual nutrients. Instead, the structure of foods, the presence of other constituents, their internal organization, and even the dietary context in which they are consumed are also crucial for understanding their health impact [4,5].

Central to this evolution is the emerging concept of the food matrix, which underscores the notion that the nutritional and health impacts of a food item extend beyond, in unpredictable ways, the mere sum of its individual nutrients, referred to as the food matrix’s health effects. In 2017, an eminent group of researchers acknowledged that the health effects of a particular food are much more complex than that of single nutrients or even a few nutrients, owing to this food’s matrix [6].

The role of the food matrix in health outcomes seems to occur due to differences in the digestion, absorption, and bioavailability of nutrients within different foods. Research suggests that, e.g., the physical structure of a food significantly influences how the compounds are digested, released, and absorbed from its matrix in the gastrointestinal tract upon consumption [7]. This effect has significant implications for physiological responses to foods and their nutrients and, consequently, the risk of disease. Indeed, an expanding body of both in vitro and clinical research suggests that nutrients consumed within a food matrix, rather than in isolation, can meaningfully affect factors such as the postprandial lipid and protein profiles [7]. The effect of the food matrix has been reported in different foods such as grains [8] and fruits [9]. For instance, despite almonds’ high lipid content (50–55%), a meta-analysis of randomized controlled trials shows that they do not increase body weight or body mass index [10], likely due to low lipid bioaccessibility, attributed to the almond cell walls’ structural integrity, which protects lipids from digestion in the digestive tract [11]. This also depends on the degree of processing and particle size [12].

## 2. Dairy Foods as Examples of Food Matrices: The Dairy Matrix

**Figure 1:**
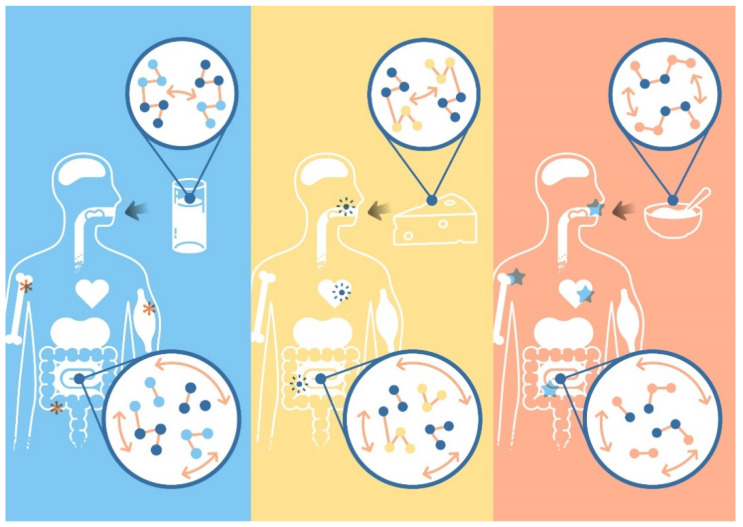
The impact of dairy matrices on health: This graph illustrates the effects of different dairy matrices (milk—blue panel, cheese—yellow panel, and yogurt—orange panel) on human health, focusing on the digestive process and nutrient absorption. Each panel shows how specific dairy products have different matrices with different components, structures, and interactions. The digestive system diagrams in each panel indicate how the different matrices of these dairy products influence nutrient digestion and absorption. This, in turn, exerts different health benefits within the body, which is illustrated by the different symbols in the human body.

The importance of considering the food matrix is clearly illustrated by dairy foods. Dairy foods contain a variety of nutrients, including minerals, vitamins, fats, lactose, and proteins, with some also containing probiotics [13]. The interaction between the components within a dairy food and the structure they form leads to a dairy matrix. Milk is an emulsion consisting of fat droplets suspended in an aqueous phase containing proteins, vitamins, and minerals [14]. The composition and structure of cheese, yogurt, and other dairy products vary depending on the type of milk used and the production method [15]. Different structural levels can be identified within a dairy matrix: the molecular level (e.g., the quaternary, tertiary, secondary, and primary structure of proteins), microscopic level (e.g., the protein network), and macroscopic level (e.g., texture). These unique variations in structure and interactions create defined matrices that modulate the extent and kinetics of nutrient digestion, influencing physiological responses [6]. For instance, clinical data showed that specific dairy systems with different macrostructures (liquid vs. semi-solid) and same caloric contents cause different satiety responses [16]. Figure 1 illustrates this concept of how various dairy matrices—milk, cheese, and yogurt—differ in their interactions and structures, impacting nutrient digestion and absorption differently and leading to varied physiological effects.

Milk and dairy products are included in almost all food-based dietary guidelines worldwide [17]. The key role of dairy foods in human nutrition has generally been attributed to their nutrient richness. They are also sources of saturated fatty acids and sodium, which are nutrients associated with negative health effects [18]. However, it appears that the impact of dairy foods on health extends beyond their individual nutrients and results in unanticipated health effects. Research has consistently shown that the consumption of various forms of dairy foods has either favourable or neutral associations with cardiometabolic health outcomes, including CVDs and type 2 diabetes (T2D). For instance, Giosuè et al. [19] showed that total dairy consumption was not associated with CVDs and indicated that the effect on cardiovascular health appears to depend more on the food type (cheese, yogurt, milk) than on the fat content. This has been supported by a recent review [20]. A neutral association was found for milk, while fermented products were associated with a decreased risk of total mortality and cardiovascular events [19]. Other notable health effects of dairy foods include the inverse association between yogurt consumption and the incidence of T2D [21,22,23], the inverse association of dairy intake and colorectal cancer [24,25,26], and the beneficial effects of dairy intake on bone health [27,28,29]. These, sometimes counter-intuitive, beneficial health effects of dairy foods (despite their saturated fat content) can be attributed to the overall dairy matrix (definitions will be provided in Section 4). The health effects of the milk, cheese, and yogurt matrices are described in more detail by the International Dairy Federation (IDF)’s technical factsheets [30,31,32].

Several components within the dairy matrices may play a crucial role in eliciting beneficial biological responses. Recently, Torres-Gonzalez and Rice Bradley [33] described the potential mechanisms underlying the beneficial effects of whole milk dairy foods on risk markers for cardiometabolic health. Key factors include the role of calcium and the varied composition of dairy fat resulting in a lowered risk of development of cardiovascular-metabolic diseases [34]. Moreover, certain compounds within the dairy matrix might amplify the health benefits of dairy. For instance, the fermentation process of yogurt and cheese yields unique bioactive compounds, such as short-chain fatty acids and peptides produced by bacteria, which have been shown to contribute to improved insulin sensitivity and reduced blood pressure [35,36].

The mechanisms of the health effects from dairy foods are not yet fully understood, since many different mechanisms are involved simultaneously, but they are usually related to the combination and interactions of the nutrients in a defined food structure, which is referred to as the dairy matrix effect in the case of dairy products. A visual representation of the dairy matrix and its health effects can be seen in Figure 1. The dairy matrix is a complex area, and more research is needed to fully understand the different dairy matrices and their different effects on health. 

## 3. The Application of the Food Matrix and Dairy Matrix Concepts in the Literature

**Figure 2:**
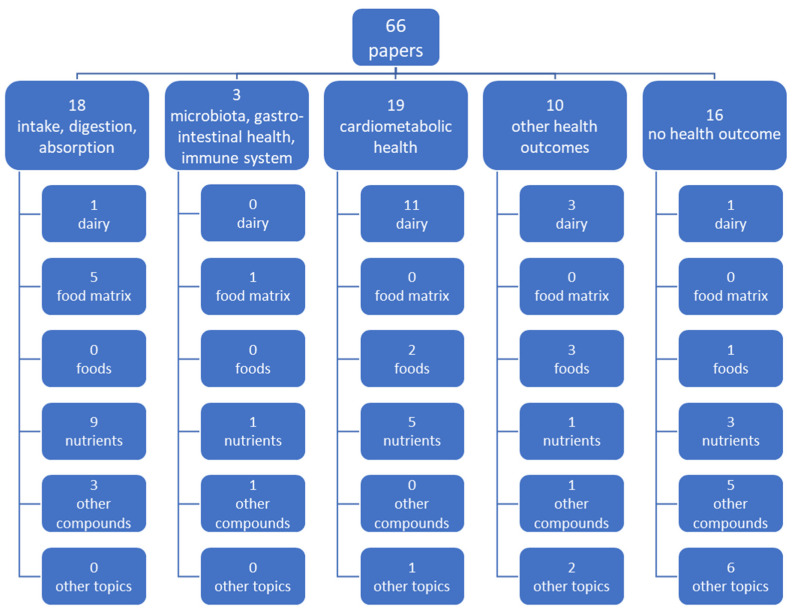
Map of the applications of the food matrix in the literature.

A move to a more holistic view on nutrition and health has introduced the concept of the food matrix among the nutrition science community. This section aims to provide an overview of how the food matrix concept has been applied in relation to nutrition and health. Using a PubMed search with the terms “food matrix”, “nutri*”, and “health” and searching for publications from the past 10 years, a map of the literature was derived. This mapping was not intended to provide a comprehensive literature review but to provide a general idea of how the concept of the food matrix is being used in nutrition science. Figure 2 presents a map of the literature using the term food matrix in the title and/or abstract in relation to human nutrition and/or health. The first level of classification referred to the outcomes of the studies and included the following categories: (1) intake, digestion, and absorption; (2) microbiota, gastrointestinal health, and immune system; (3) cardiometabolic health; (4) other health outcomes; and (5) no health outcomes. A second set of categories was added to classify the papers based on their main component of investigation and included the following: (1) dairy matrix or dairy foods; (2) food matrix; (3) foods (e.g., specific non-dairy foods); (4) nutrients (e.g., protein, lipids, fatty acids, minerals); (5) other bioactive compounds (e.g., phenolic compounds, probiotics, prebiotics); and (6) other topics (e.g., food processing techniques, fortification, supplementation, dietary recommendations).

When analysing the information in the papers retrieved, two important features of the food matrix concept emerged: (1) the food matrix influences the metabolic fate of nutrients and subsequently the health effects of these nutrients; and (2) food processing affects the food matrix and hence modulates the relation between the nutrients and health.

Based on the applications in which the food matrix concept was used, the food matrix may be conceptualized as a modulator of the theoretical health effects of nutrients: the food matrix influences nutrient intake, digestion, and absorption, in turn leading to health effects that differ from what can be expected based on the nutrient composition alone. In the words of Fardet and Rock [37], “[…] two foods with identical compositions but differing structures (i.e., food matrices) may have different metabolic and physiological effects in the short term and health effects in the long term.”

The majority of studies in our review focused on dairy foods and/or saturated fatty acids. The dairy matrix is put forward as an explanation for the discrepancy between the theoretical health effects and observed health effects. From the reviewed literature, there is a wide range of functionalities associated with the dairy matrix, which includes cardiometabolic health, digestion, bone and muscle health, and appetite. However, the majority of the publications focused on cardiometabolic health, particularly the role of saturated fatty acids.

Interestingly, several studies describe dairy matrix health effects without referring to the concept of the dairy matrix. We may conclude that the concept of the dairy matrix generally refers to the combination and interaction of nutrients within a food structure. However, there is a need for a clear and harmonized definition.

## 4. Definitions of the Food and the Dairy Matrices

To build a definition of the concept of the dairy matrix, one must first consider the broader term of the food matrix. Analysing scientific articles from the past 10 years, we found that some publications provided a definition or relevant snippets that gave some understanding of this concept.

Some of the authors related the concept of the food matrix to the nature of the food [6] and defined it as whole foods [38,39]. Other authors highlighted the complexity of the concept and that it goes beyond the traditional approach of considering individual nutrients alone [22,40,41].

Several authors mentioned interactions between nutrients [40], between nutrients and the structure of a food [42], and how nutrients, structure, and spatial organization interact with each other [43]. In that regard, Donovan and Goulet [44] discussed molecular relations, while Cifelli [45] highlighted the interactions between food attributes.

Some definitions included more general effects, for example “modulating the properties and metabolism of any single nutrient it contains” [46], that it can “influence the digestion of food, the absorption of nutrients and bioactive compounds, and the physiological functions that impact health” [45], and that it “can modulate food digestion and subsequent nutrient absorption … Consequently, this food matrix can strongly affect the metabolic impact of consumed nutrients.” [47].

From Table A1 in Appendix A, it can be seen that there are three themes that are particularly repeated, i.e., nutrients, structure, and interactions. Nutrients and structure are repeated with different terminology, as illustrated in Table A2 in the Appendix A.

Compared to the term “food matrix”, there were fewer definitions of the term “dairy matrix” within the literature (see Table 1). Extracts suggested a potential definition rather than a specific definition. Some publications referred to the general concept of the food matrix. Some authors highlighted that dairy foods are considerably different in the complexity of their food matrices [43,46,48,49]. This is due to the different production methods, which change the content and type of nutrients, structures, and how they interact. Some authors have referred to the existence of three main types of dairy matrices [44,50], liquid (most often illustrated by milk), semi-solid or gel (e.g., yogurt), and solid (e.g., cheese).

Based on this literature review, it is evident that a precise definition of the term “food matrix” is lacking, despite its growing use in the area of nutrition and health. Additionally, the term “dairy matrix” does not seem widely used within a specific scientific or technical framework.

Having a harmonized definition of the dairy matrix that is globally recognized would promote clarity and efficiency, ensuring consistency and unified communication. The International Dairy Federation is the world’s leading source of expertise and scientific knowledge for the dairy sector. Expert members of its nutrition and health committee developed the following definitions, which aim to balance the complexity of the concepts and their practical application (it is important to note that a similar definition can be made for the food matrix and food matrix health effects).

→ **The “dairy matrix” describes the unique structure of a dairy food, its components (e.g., nutrients and non-nutrients), and how they interact.**
→ **“Dairy matrix health effects” refers to the impact of a dairy food on health that extends beyond its individual components (e.g., nutrients and non-nutrients).**

## 5. Implementing the Food/Dairy Matrix Concept into Nutrition Policies and Practices

Current nutrition strategies aiming for improved public health are based on nutrient profiles. This perspective has led to the classification of foods based on their isolated nutrient content, detached from the carrying food product, resulting in for example the development of front-of-pack nutrition labelling (FOPNL) schemes. These schemes have been developed as a tool to assist consumers in making healthier food choices by providing clear information about the nutritional content of products. However, FOPNL labels only evaluate a limited amount of nutrients, independently of food origin/type. Hence, they are unable to comprehensively evaluate the effect of foods, meals, and dietary patterns on health, which can lead to unintended consequences such as the discouragement of nutrient-dense foods [56].

As the scientific community continues to unravel the complexities of the food matrix, including the dairy matrix, opportunities arise to integrate these insights into the space of dietary recommendations, food policies, and product labelling. Scientific evidence underscores the importance of expanding future public health initiatives beyond the confines of nutrient content in FOPNL schemes. The current evidence shows the interplay among nutrients and the overall food carrying them in relation to nutrition and health. These findings strongly advocate for a shift towards incorporating the concept of the food matrix, rather than solely focusing on individual nutrients, into the development of food-based nutrition guidance. A meaningful step forward could be establishing an FOPNL food group, since that would allow meaningful thresholds to be applied to different foods with different food matrices—e.g., different saturated fatty acid thresholds for dairy vs. biscuits and cakes. 

In this context, it is also crucial to note the distinction between different types of nutrients within the same classification, e.g., naturally occurring trans-fat (also referred to as ruminant trans-fat) and industrially produced trans-fat. In contrast to industrially produced trans-fat, the consumption of ruminant-derived trans-fat at doses achievable by the diet alone has no adverse effect on blood lipids or other coronary heart disease [57,58,59]. Furthermore, evidence shows that the biological activities of industrial and ruminant trans-fatty acids differ, and that certain ruminant trans-fatty acids (such as rumenic, vaccenic, and trans-palmitoleic acids) may be associated with beneficial health effects in humans [60,61].

As mentioned previously, the effects of dairy foods on human health cannot be predicted solely by their nutrient content, which seems to be especially true for their saturated fat, sodium, and naturally occurring sugar content. Reductionist nutritional strategies that focus solely on individual discouraged nutrients detached from the food matrix have been shown to lead to unintended consequences related to nutritional adequacy and diet quality [62]. In the case of dairy foods, the nutrients in dairy foods and their bioavailability make it challenging to replace dairy with other foods, which are often also less affordable [63]. Rehm and Drewnowski [64] found that removing 5% of dietary energy from dairy fat coming from whole milk dairy foods, milk, cheese, and yogurt and replacing it with foods containing a comparable amount of non-dairy polyunsaturated fatty acids and monounsaturated fatty acids led to lower amounts of multiple nutrients in the diet, such as calcium, vitamin D, vitamin A, riboflavin, niacin, and vitamin B12, resulting in a lower diet quality.

Therefore, policy actions should evolve to emphasize not only individual nutrient intake but also the consumption of foods and dietary patterns that optimize the health benefits derived from their unique matrices. Tailoring recommendations based on the food matrix could lead to more personalized and effective dietary advice.

Finally, the above should be considered within a plan for future initiatives in nutrition education. This could leverage the food matrix and dairy matrix concepts to communicate the broader health implications of dietary choices. Continuous research and dedicated public health endeavours are essential, emphasizing the importance of gaining a more comprehensive understanding of the health properties associated with the matrices of foods in general and of dairy foods. Exploring how these foods fit within overall dietary patterns, considering individual characteristics and needs, is crucial. These initiatives are pivotal in advancing guidance that effectively reduces the burden of diet-related diseases and enhances global public health.

## 6. Conclusions

The concept of the food matrix and especially the dairy matrix has received much attention lately in reference to its effects on nutrition and health. However, the term “dairy matrix” is used vaguely or not at all by food and nutrition scientists, often as synonymous with the specific dairy food itself or its structure. This is clearly illustrated by the number of publications related to the dairy matrix that our PubMed search could not pick up. Therefore, we urge the scientific community to spread the use of the terms “food matrix” and “dairy matrix” in the titles and/or abstracts of scientific publications. The definitions developed by the IDF may help with international harmonization of the use of dairy matrix and dairy matrix health effects and may also be extrapolated onto the food matrix and food matrix health effects.

Integrating food and dairy matrix concepts into dietary guidelines, policies, and labelling practices promises a new era of evidence-based, holistic nutrition. As our matrix understanding deepens, stakeholders can collaborate to shape policies promoting health for individuals and populations.

## Figures and Tables

**Table 1:** Relevant definitions and snippets of the term dairy matrix from reviewed research from the past 10 years.

Definition/Relevant Snippets	References
“*The nutritional value of dairy products should therefore be considered as the biofunctionality of the sum of nutrients within the dairy matrix structures*”	[51]
The cheese matrix was defined as cheese’s structure	[52]
“*There are three main types of dairy food matrices: liquid (milk, some fermented milks), semi-solid (yogurt, some fresh cheeses), and solid (most cheeses).*”	[44]
*“Consumption of isolated dairy-derived nutrients* *has been found to impact the risk of cardiometabolic disease di* *fferently compared to a whole dairy matrix. Dairy products considerably differ in the complexity of their food matrix due to processing methods (e.g., milk vs. cheese), which alters their nutrient types and composition, as well as physical* *structure.”*	[46]
“*While dairy products are often considered together as a food category in nutritional epidemiology, they vary considerably in terms of their content and structure and how these interact with other food components, which describes the ‘dairy matrix’ concept.*”	[49]
“*The health impact of dairy fats and dairy foods must take into account their complex matrix (e.g., milk oligosaccharides, calcium, live and active cultures in yogurt, milk fat globule membranes and polar lipids, and bioactive peptides), which contribute to the gastrointestinal (GI) tract milieu of diet-derived factors that influence the host and microbiome.*”	[48]
“*Dairy food matrix, i.e., the specific nutrient mix and the physical structure they sit within, and appreciate how this differs across different types of dairy foods, e.g., milk vs. yoghurt vs. cheese.*”	[43]
“*The dairy matrix is not only the composition of nutrients, bioactive constituents, and other compounds present in milk and other dairy products but also how they are packaged and compartmentalized. It reflects the processing that the product undergoes, including changes in physical state of the product, altered endogenous constituents, and addition of inert and live chemicals or microorganisms.*”	[53]
“*La matriz láctea está compuesta por lípidos, proteínas, hidratos de carbono, minerales y otros componentes minoritarios, cuya interacción determina no solo los aspectos sensoriales de estos alimentos, sino sus propiedades nutricionales y sanitarias.*” Translation: “*The dairy matrix is composed of lipids, proteins, carbohydrates, minerals and other minority components, whose interaction determines not only the sensory aspects of these foods, but also their nutritional and health properties.*”	[54]
“*The dairy matrix, comprising both solid matrices (cheese), semi-solid matrices (yoghurt, crème fraiche), and liquids (milk, cream), receives attention. In vitro and in vivo studies have indicated that the physical structure and processing of dairy products may affect bioavailability of nutrients.*”	[50]
“*The dairy matrix is the structural organization of physically and chemically interacting components of dairy products that influence nutrient bioaccessibility, nutrient bioavailability, gut endocrine function and/or gut microbiota. (Bioaccessibility: release of nutrients from the food matrix)*”	[55]

## Appendix A

**Table A1:** Relevant definitions and snippets of the term food matrix from reviewed research from the past 10 years.

Definition/Relevant Snippets	References
Some aspects of a food matrix: “*the understanding of digestion mechanisms of major nutrients gained from the studies that use individual ingredients will be useful in reaching another level of complexity—the microstructural organizationof a real food matrix.*”	[65]
“*Food matrix effect is the difference between the effects of a sum of nutrients and those of a food. It reflects the fact that beyond nutrients there is non nutritive components, physico-chemical structures, interactions between nutrients, different bioavailability, which may explain some specific effects of foods, particularly when they are whole or not refined foods.*”	[40]
“*The nature of the food structure and the nutrients therein (i.e., the food matrix)*”	[6]
“*The matrix effect of a food goes beyond the individual nutrients, suggesting that the physical structure, created by a combination of nutritive components, can act independently of its individual components during digestion and metabolism*”	[22]
“*The food matrix describes foods in the context of both their structure, and their nutrient content, with the goal of understanding how these interact together*”	[42]
The food matrix is referred to as “*the nutrient and nonnutrient components of foods and their molecular relations*.”	[44]
The food matrix is defined as whole foods	[38]
The food matrix is defined as whole foods	[39]
The food matrix is described as “*the physical domain that interacts with specific constituents of a food*”	[7]
“*The food matrix, in essence the entire structure and composition of nutrients consumed by an individual, is gaining scientific recognition for its role in modulating the properties and metabolism of any single nutrient it contains.*”	[46]
The food matrix is defined as “*the food structure and nutrients therein*”	[66]
“*Overall composition and structure of foods needs to be considered, i.e., the food matrix effects.*”	[67]
“*The term ‘food matrix’ therefore describes the overall structure of a food, the spatial organisation of the nutrients and structures within it, and how these interact with each other.*”	[43]
“*This concept—referred to as the food matrix—considers all the attributes of food, including its microstructure, texture, and form (e.g., solid, gel, liquid), and how the nutrients and bioactive compounds are packaged and compartmentalized. Collectively, these attributes interact in ways that influence the digestion of food, the absorption of nutrients and bioactive compounds, and the physiological functions that impact health.*”	[45]
“*Whole food, i.e., its form and matrix, beyond the traditional approach of its individual nutrients and bioactive constituents*”	[41]
Some relevant snippets: “*evidence is increasing that the nutritional properties of a food ares not only determined by its single nutrients, but also by the complex food structure in which the nutrients are embedded. Thus, studies indicate that the food matrix can affect digestion and absorption of the nutrients in a given food.*”	[50]
“*Whole-food products contain multiple nutrients incorporated in a complex structure that can modulate food digestion and subsequent nutrient absorption, which is referred to as the food matrix. Consequently, this food matrix can strongly affect the metabolic impact of consumed nutrients.*”	[47]

**Table A2:** Analysis of how the concepts of nutrient and structure are used in the definitions of the food matrix.

Concept	Specific Terms Used	References
Nutrient	nutrients therein	[6,66]
	non-nutrients	[40]
	nutrient and non-nutrient components	[41]
	nutrient content	[42]
	overall composition	[67]
	nutritive components	[22]
	composition of nutrients	[46]
	nutrients and bioactive compounds	[45]
	multiple nutrients	[47]
Structure	food structure	[6,42,66]
	overall structure	[43,67]
	entire structure	[46]
	complex structure	[47]
	structure within	[43]
	physical structure	[22]
	physico-chemical structure	[40]
	microstructure	[45]
	texture	[45]
	form (e.g., solid, gel, liquid)	[45]
